# Human access and deterministic processes play a major role in structuring planktonic and sedimentary bacterial and eukaryotic communities in lakes

**DOI:** 10.7717/peerj.14378

**Published:** 2022-11-11

**Authors:** John K. Pearman, Georgia Thomson-Laing, Lucy Thompson, Sean Waters, Marcus J. Vandergoes, Jamie D. Howarth, Ian C. Duggan, Ian D. Hogg, Susanna A. Wood

**Affiliations:** 1 Cawthron Institute, Nelson, New Zealand; 2 GNS Science, Lower Hutt, New Zealand; 3 Victoria University of Wellington, Wellington, New Zealand; 4 University of Waikato, Hamilton, New Zealand; 5 Canadian High Arctic Research Station, Nunavut, Canada

**Keywords:** Metabarcoding, eDNA, Lakes, Plankton, Sediment, Bacteria, Eukaryotes, Deterministic processes

## Abstract

Lakes provide habitat for a diverse array of species and offer a wide range of ecosystem services for humanity. However, they are highly vulnerable as they are not only impacted by adverse actions directly affecting them, but also those on the surrounding environment. Improving knowledge on the processes responsible for community assembly in different biotic components will aid in the protection and restoration of lakes. Studies to date suggested a combination of deterministic (where biotic/abiotic factors act on fitness differences amongst taxa) and stochastic (where dispersal plays a larger factor in community assembly) processes are responsible for structuring biotic communities, but there is no consensus on the relative roles these processes play, and data is lacking for lakes. In the present study, we sampled different biotic components in 34 lakes located on the South Island of New Zealand. To obtain a holistic view of assembly processes in lakes we used metabarcoding to investigate bacteria in the sediment and surface waters, and eukaryotes in the sediment and two different size fractions of the water column. Physicochemical parameters were collected in parallel. Results showed that deterministic processes dominated the assembly of lake communities although the relative importance of variable and homogeneous selection differed among the biotic components. Variable selection was more important in the sediment (SSbact and SSeuks) and for the bacterioplankton (Pbact) while the assembly of the eukaryotic plankton (SPeuks, LPeuks) was driven more by homogeneous selection. The ease of human access to the lakes had a significant effect on lake communities. In particular, clade III of SAR11 and *Daphnia pulex* were only present in lakes with public access. This study provides insights into the distribution patterns of different biotic components and highlights the value in understanding the drivers of different biological communities within lakes.

## Introduction

Lakes are often considered sentinels of environmental change as they accumulate nutrients and environmental contaminants from the surrounding landscape and atmosphere (Williamson et al., 2008; Adrian et al., 2009). The important and diverse ecosystem services provided by lakes (*e.g*., food and recreation) mean that humans have utilised lakes and their catchments for millennia. Over this time, lakes have been subjected to multiple stressors including catchment land use modification, increased nutrients, anthropogenic pollution and the introduction of non-indigenous species (Dudgeon et al., 2006). These stressors have had profound effects on lake biota and resulted in a global decline of lake ecosystem health (Arbuckle & Downing, 2001; McCrackin et al., 2017). To ensure appropriate protection and limit the degradation in lake health, it is vital to understand how biotic components are responding to stressors and how this varies amongst lakes.

Lakes are ideal for studying biogeographic patterns as they are discrete bodies of water isolated from each other by terrestrial habitats. Even over relatively small spatial scales, lakes can vary in size, depth and geomorphology, and in the land use and geology of their surrounding catchments (Wetzel, 2001). These inherent features of lakes provide an ideal opportunity for examining differences in biotic communities and drivers of biodiversity among lakes. The water column and sediments are two main habitat types within lakes which are characterised by distinctive environmental properties, but which are in continuous contact and interconnected (Forsberg, 1989). Planktonic communities of lakes have received substantial research (Drakare & Liess, 2010; Newton et al., 2011; Liao et al., 2017; Yang & Zhang, 2020; Pearman et al., 2020; Kraemer et al., 2020; Schallenberg et al., 2021; Duggan, Özkundakci & David, 2021). In contrast, sediment communities have received comparatively less attention (Zeng et al., 2019; Pearman et al., 2020; Jiao et al., 2020) despite sediments acting as a sink for organic matter, nutrients and pollutants. Biological communities present within the sediments are also vital in many biogeochemical processes (Forsberg, 1989). Studies investigating community patterns in both the water column and sediments are notably fewer and focused mostly on differences in bacterial communities between the two habitats (Zeng et al., 2019; Jiao et al., 2020, 2021; Xia et al., 2022).

Various ecological theories have been developed to understand how communities are assembled. Niche theory highlights the importance of deterministic processes and indicates that species can co-exist by occupying different niches (Levine & HilleRisLambers, 2009). Hubbell’s neutral theory in contrast describes the importance of stochastic processes such as birth/death and colonization and extinction (Hubbell, 2001). Investigations of ecosystems have suggested a combination of deterministic and stochastic assembly processes are responsible for structuring biotic communities (Chase, 2010; Stegen et al., 2013; Zhou & Ning, 2017; Sadeghi et al., 2021), however, there is no consensus on the relative roles these processes play (Vellend et al., 2014; Stegen et al., 2015; Zhou & Ning, 2017). The basis of deterministic processes is that there are differences in fitness amongst taxa that result in varied reproductive/replication rates (Vellend, 2010). Deterministic processes can be classified into either variable or homogeneous selection. Variable selection occurs when environmental conditions vary spatially and/or temporally and selective pressures result in high variation in the community structure (Zhou & Ning, 2017). In contrast, homogeneous selection happens when there is little variation in the environmental conditions leading to a more similar community structure than would be expected (Zhou & Ning, 2017). Previous studies have suggested that within lakes, abiotic factors such as pH, temperature and nutrient conditions (Hoostal & Bouzat, 2008; Ruuskanen et al., 2018; Pearman et al., 2020) and catchment characteristics, such as land use and altitude (Hayden & Beman, 2016; Li et al., 2017; Pearman et al., 2020; Kraemer et al., 2020) can influence biotic communities. Further, biotic interactions such as predation and competition can also have deterministic effects on communities (Zhou & Ning, 2017). Stochastic processes can be relevant when taxa are functionally equivalent and environmental factors are not strongly influencing community assembly (Sloan et al., 2006; Drakare & Liess, 2010). Stochastic processes can be divided into dispersal limitation and homogenizing dispersal. Dispersal limitation occurs when there are barriers to the effective transfer of organisms between communities, coupled with ecological drift leading to increased variations between communities (Stegen et al., 2015). In contrast, homogenizing dispersal occurs when there is a high level of dispersal/gene flow between communities resulting in communities that are more similar than would be expected by chance alone (Zhou & Ning, 2017). Stochastic processes could be impacted by the access humans have to lakes, with anthropogenic activities being implicated for spreading non-indigenous species (Craig, 1992; Mills et al., 1994; Rowe, 2007; Kilroy et al., 2021).

Until recently, assembly processes have received limited attention in lakes and studies have mainly been restricted to bacterial communities. In general, deterministic processes have been shown to dominate the structuring of biotic communities (Liao et al., 2017; Logares et al., 2018; Zeng et al., 2019; Jiao et al., 2020, 2021; Kraemer et al., 2020). In the water column, homogeneous selection was found to be dominant for bacteria in research ranging from a large scale study of Canadian lakes (Kraemer et al., 2020) to more restricted spatial scales in China (Zeng et al., 2019; Jiao et al., 2020, 2021) and Antarctica (Logares et al., 2018). However, Jiao et al. (2020) showed that assembly processes were seasonally variable. For eukaryotes, Logares et al. (2018) could not identify a dominant process responsible for structuring communities in Antarctic lakes, with the authors suggesting the community differences were a result of ecological drift. They also highlighted that different biological taxonomic groups in the same lakes could be structured by different processes. While investigations are limited, lake sediment bacterial communities have been shown in a variety of Chinese lakes to be predominantly assembled by variable selection (Zeng et al., 2019; Jiao et al., 2020, 2021; Xia et al., 2022). In general, there is a pattern of homogeneous selection in the water column and variable selection in the sediment (Zeng et al., 2019; Jiao et al., 2020; Xia et al., 2022). This is most likely due to variability in the sediment structure, as well as the accumulation of nutrients and pollutants from the catchment making the sediment more heterogenous.

To enhance understanding of the distribution patterns of bacteria and eukaryotes in the water column and sediment of lakes, we sampled different biotic components in 34 lakes located on the South Island of New Zealand. We targeted bacteria in both the surface sediment and water, as well as eukaryotes in the surface sediment and two different size fractions of eukaryotes in the water column to obtain a more holistic view of assembly processes in lakes. The lakes sampled varied along a gradient of human access enabling us to determine if this influenced biotic communities. We hypothesised that: (1) each biotic component would have different assembly processes; (2) communities in the sediment would have a higher contribution from variable selection than those in the water column which would be predominantly assembled by homogeneous selection; and (3) that lakes with human access would have different communities to those with no access, because humans act as a mechanism for dispersal.

## Methods

### Study lakes

**Figure 1:**
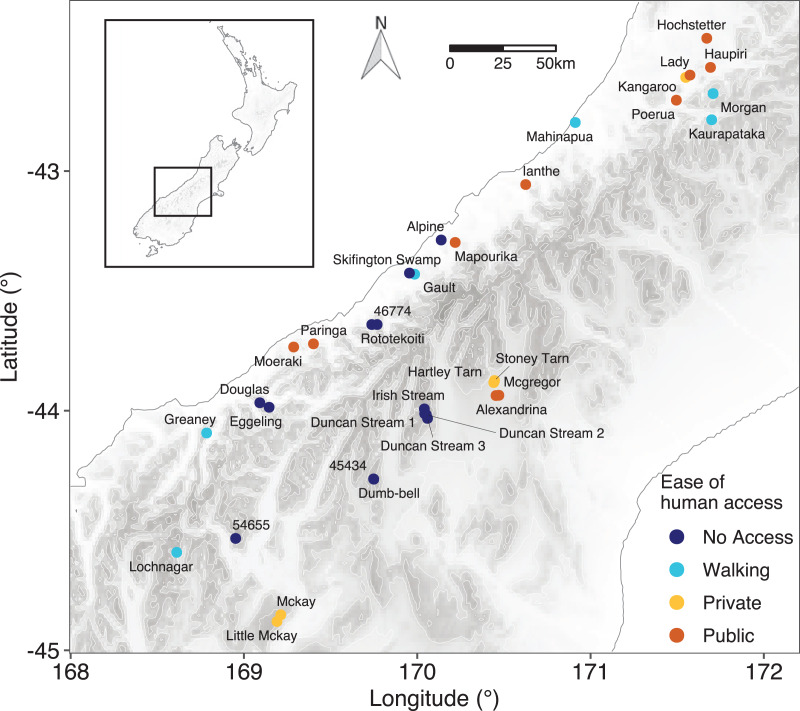
Location of the lakes sampled. Details of sampling positions and lake characteristics are given in Table S1. Points are coloured based on the classification of ease of human access to the lake.

A total of 34 lakes were sampled on the central and western side of the South Island of New Zealand between 21 January 2020 and 14 February 2020 (Fig. 1, Table S1). Lakes included a range of altitudes from 9 m (Lake Ianthe) to 1,839 m (Duncan Stream 1) above sea level.

Descriptions of the land cover in the catchments were derived from the most up to date satellite imagery available in the Land Cover Database Version 5 (Landcare Research New Zealand Ltd: https://lris.scinfo.org.nz/layer/104400-lcdb-v50-land-cover-database-version-50-mainland-new-zealand/). Eight broad groupings were used for the analysis and were: (1) native vegetation; (2) urban; (3) non-indigenous vegetation; (4) water; (5) forestry; (6) high production grassland; (7) low producing grassland; and (8) other (see Table S1 for details of these groups).

Lakes were classified into four categories based on the ease of human access to the lake: (1) no easy access, where the lakes were in a remote location with no public or formed access tracks; (2) walking access, where there was a public walking/hiking track to the lake but no vehicle access; (3) private access, where access was possible by vehicle but restricted due to the lake being on private land; and (4) public access, where the lake was publicly accessible by vehicle.

All samples were collected under the specifications of Special Permit 651 from the New Zealand government agency Ministry for Primary Industries.

### Chlorophyll and nutrient analysis

Surface water for chlorophyll *a* analysis was collected in triplicate sterile bottles (500 mL) from a single site at the deepest point in the lake. A subsample (~200 mL from each bottle) was filtered through a single GF/C filter (combining to ~600 mL) before being wrapped in aluminium foil and stored at −20 °C. Analysis was undertaken at the Watercare Laboratories (Auckland, New Zealand) following the APHA 10 200H method with a reporting limit of 0.0006 mg/L.

Water samples (1 L) were collected using an integrated tube sampled through the surface mixed layer (as determined by RBR profiles, RBR Ltd, Ottawa, Canada). Total nutrient analysis was undertaken on a flow injection analyzer using American Public Health Association (APHA) 4500 methods (https://www.standardmethods.org/). Reporting limits for total nitrogen (TN) and total phosphorus (TP) analyses were 0.005 mg/L. Combustion analysis at 850 °C using APHA 5310 B methods (https://www.standardmethods.org/) was used for determination of total organic carbon (TOC) with reporting limits of 0.5 mg/L.

Samples for analysis of sediment biogeochemistry were collected from a single site at the lake depocenter. Three ponar grab samples were taken and sediment collected from the top 2 cm. These were combined and stored in 500 g containers, which were chilled at 4 °C and shipped to the laboratory within 48 h. Samples were homogenized, centrifuged (3,000×*g*, 40 min, 4 °C) and the pore water decanted. Following the removal of carbonates by acid pre-treatment, TN and TOC were analyzed using catalytic combustion at (900 °C, O_2_) and separation using a Thermal Conductivity Detector (reporting limit for both g/100 g sediment). Total P was measured in the sediment by drying and sieving the sediment before acid digestion followed by Inductively Coupled Plasma-Mass Spectrometry (ICP-MS) analysis based on the US Environmental Protection Agency (EPA) method 200.8 with a reporting limit of 10 mg kg^−1^.

### Biological sample collection and processing

Surface water samples were collected in triplicate (500 mL) sterile bottles that had been rinsed three times with lake water. Bacterioplankton (Pbact) were filtered onto a 0.22 µm S-Pak filter (Millipore Sigma, Burlington, MA, USA) using a volume of 100 mL per replicate and stored at −20 °C until later DNA extraction.

Small and large planktonic eukaryotes (SPeuks and LPeuks, respectively) were collected using a 20 and 40 µm mesh plankton net, respectively. Vertical trawls from ~1.5 m above the lakebed were used except in Stoney Tarn which, due to its shallow depth, was sampled by pouring 10 L of surface water through the nets. The concentrated biomass of each trawl was transferred to separate 50 mL Falcon tubes and preserved with 95% ethanol.

A ponar grab sampler was used to collect surface sediment samples for both bacteria (SSbact) and eukaryotes (SSeuks). Samples (1 g) were taken from the undisturbed surface layer (top 5 mm) using a sterile spatula and stored in LifeGuard™ Soil Preservation Solution (Qiagen, USA) and stored at −80 °C for later DNA extraction.

Molecular analysis (*i.e*., DNA extraction, Polymerase Chain Reaction (PCR) set-up, template addition and PCR analysis) were conducted in separate sterile laboratories dedicated to each step to reduce potential cross contamination. Prior to DNA extraction, amplification set-up and template addition the laboratories were UV sterilized for 15 min. PCR set-up and template addition were undertaken in laminar flow cabinets fitted with HEPA filters. For all steps aerosol barrier tips (Eppendorf, Germany or Axygen, United States) were used.

DNA extractions were undertaken using the DNeasy PowerSoil Kit (Qiagen, Hilden, Germany). For the Pbact samples, filters were cut into five pieces using sterile techniques and placed in the PowerBead tubes of the kit. For both the SPeuks and LPeuks samples, biomass was pelleted by centrifugation (3,000×*g*, 20 min, 4 °C) and added to the PowerBead tubes. For the surface sediment samples, a subsample (0.25 g) was placed in the PowerBead tubes. All further steps were as per the DNeasy PowerSoil Kit instructions using a QIAcube. Negative extraction controls were included after every 23 samples.

Bacteria, in the surface waters (Pbact) and surface sediment (SSbact) were targeted by primers for the V3-V4 regions of the bacterial 16S ribosomal RNA gene (16S rRNA; Table S2; Herlemann, Geissinger & Brune, 2007; Klindworth et al., 2013). The SPeuks samples as well as the SSeuks were amplified by primers for the 18S ribosomal RNA gene (18S rRNA; Table S2; Zhan et al., 2013). The LPeuks were assessed using mitochondrial cytochrome oxidase I (COI) primers as this gene has good representation of zooplankton in reference databases (Table S2; Leray et al., 2013). Illumina™ overhang adaptors were attached to the taxa specific primers to allow for dual-indexing (Kozich et al., 2013). PCR reactions were undertaken in triplicate 20 µL volumes and contained 10 µL of MiFi 2 × PCR mastermix, 2 µM of each primer (final conc.) and 2 µL of template DNA. All PCRs had an initial denaturation step of 94 °C for 5 min and a final extension time of 5 min at 72 °C. The cycling conditions for each primer are given in Table S2. Negative controls for both the PCR and extraction were included. SequalPrep normalization plates (ThermoFisher Scientific, Waltham, MA, USA), were used to clean and normalize pooled triplicate PCR products. An Illumina Miseq™ platform, at the Auckland Genomics Facility was used for sequencing. Sequence library preparation was undertaken according to the Illumina 16S metagenomics library prep manual (https://support.illumina.com/documents/documentation/chemistry_documentation/16s/16s-metagenomic-library-prep-guide-15044223-b.pdf) with the exception that after the indexing PCR, 5 μL of each sample (including water samples acting as sequencing blank) was pooled and a single clean-up was undertaken. Quality control on the library pool was assessed using a bioanalyzer before dilution (to 4 nM), denaturation, and a further dilution to 7 ρM with a 15% PhiX spike took place.

### Bioinformatics

Bioinformatic pipelines for the three genes were identical unless otherwise stated. Cutadapt was used to remove primer sequences from the raw reads with a maximum of one mismatch allowed (Martin, 2011). Subsequent processing was undertaken using the DADA2 package (Callahan et al., 2016) within R (R Core Team, 2020). For the 16S rRNA and 18S rRNA sequence reads were truncated to 230 and 228 base pairs (bp) for forward and reverse reads respectively while for the COI gene the truncation length was 165 bp for both forward and reverse. Depending on the quality of the sequencing run, sequences were filtered with a maximum number of “expected errors” (maxEE) threshold of two or four for forward reads and four or six for reverse reads. Reads that did not match this criterion were discarded and not used for further analysis. The inference of Amplicon Sequence Variants (ASVs) was based on a parametric error matrix was constructed based on the first 10^8^ bp of the sequences. The paired-end reads were merged with a maximum mismatch of 1 bp and a required minimum overlap of 10 bp before chimeric sequences were removed using the removeBimeraDenovo script within the *DADA2* package.

For the COI dataset, pseudogenes were detected and removed using the methods described in Leray & Knowlton (2015). Translated sequences were aligned against a subset of the MIDORI database (Machida et al., 2017) using Multiple Alignment of Coding Sequences (MACSE; Ranwez et al., 2011). Sequences were first assessed using the invertebrate translation code and then subsequently the vertebrate code. Any sequences that contained a stop codon or greater than two frame shifts for both translations were considered a pseudogene and removed from further analysis.

The resulting chimera checked ASVs were used for taxonomic classification against the SILVA 138 (Pruesse et al., 2007) for the 16S rRNA, PR^2^ (Guillou et al., 2013) for the 18S rRNA and a combination of the BOLD (Ratnasingham & Hebert, 2007) and nucleotide NCBI (NCBI Resource Coordinators, 2018) supplemented with some unpublished sequences from native zooplankton for the COI gene datasets. Classification of sequences was undertaken using the rdp classifier (Wang et al., 2007) with a bootstrap of 70 and 50 for the ribosomal and COI datasets respectively, to enable classifications at higher taxonomic levels.

ASV tables can be found in Tables S3–S7.

### Statistical analysis

The ASV table, taxonomy, and metadata were combined into a *phyloseq* object (McMurdie & Holmes, 2013) for further analysis. Eukaryotic, chloroplast and mitochondrial ASVs were removed from the 16S rRNA datasets (Pbact and SSbact), while bacteria were removed from both the 18S rRNA (SPeuks and SSeuks) and COI (LPeuks) datasets. Contamination in the negative controls was assessed and read numbers for each ASV found in the negative controls were subtracted from the samples. The samples were subsampled to an even depth at a level of 24,134 reads for the SSbact, 23,188 for SSeuks, 11,710 for SPeuks, 25,297 for LPeuks and 51,362 for Pbact.

Rarefaction curves were constructed using the number of reads sequenced across all the lakes sampled for each biological component separately using the R package *ranacapa* (Kandlikar et al., 2018). Alpha diversity metrics of the observed number of ASVs and Chao1 were calculated.

Assembly processes were assessed for the biological components of the lake using ecological modelling frameworks developed and adapted by Stegen et al. (2013, 2015). Phylogenetic turnover in the biological communities between pairs of lakes was assessed by calculating the mean-nearest-taxon-distance (ßMNTD; Fine & Kembel, 2011) using the packages *msa* (Bodenhofer et al., 2015) and *phangorn* (Schliep, 2011). The phylogenetic distance was quantified between each ASV in a community and its nearest relative in a second community. A null distribution of ßMNTD was calculated using the R packages *picante* (Kembel et al., 2010) and *iCAMP* (repetitions = 999, Ning et al., 2020) using the assumption that ecological selection was not the primary cause of differences in pairs of communities. The beta-nearest-taxon-index (ßMNTI) was calculated by comparing between the null model and observed ßMNTD values and normalizing by standard deviation. Deviations away from the null model indicated that deterministic processes were prevalent, which were then allocated to one of two categories. (1) Homogenous selection, this will be dominant when environmental conditions are similar leading to consistent selective pressures and resulting in low levels of change in the community and ßMNTI values <−2. (2) Variable selection, occurs when environmental differences are sufficient to assert selective pressures on the fitness differences amongst taxa, resulting in higher than expected pairwise differences in communities and a ßMNTI >2. If pairwise comparisons did not deviate from the null distribution, then deterministic processes were likely weak and stochastic processes were then evaluated. A stochastic null model was calculated based on the Raup-Crick metric adapted to account for species relative abundances (RC_Bray_). The observed values were compared against the null model and standardised to between −1 and 1 (Stegen et al., 2015). If large differences in the community are observed while deterministic processes are low, then dispersal limitation and subsequent compositional drift of communities is determined to be the predominant processes and is indicated by RC_Bray_ >0.95. Homogenizing dispersal, where high rates of dispersal led to similar communities is indicated when deterministic processes are low and there are RC_Bray_ values <−0.95. If there are no deviations away from the null model for either the deterministic or stochastic processes, then the pairwise comparison is interpreted as having no dominant assembly process. Assembly processes were visualised using the R package *circlize* (Gu et al., 2014).

Distance similarity patterns between community similarity (1–Bray-Curtis) and environmental distance were assessed with linear regression. Environmental variables consisted of nine lake variables (TOC, TN, TP, chlorophyll *a* at the water surface, Secchi Disk depth, temperature just above the sediment, dissolved oxygen just above the sediment, lake altitude, maximum depth of the lake) and five catchment characteristics (proportion of forestry, native vegetation, non-indigenous vegetation, low productivity grassland and other (predominantly gravel)). Values for TOC, TP and TN were from the sediment for SSbact and SSeuks or from the water column for Pbact, SPeuks and LPeuks. These variables were selected after checking for co-correlation and non-zero variance using the packages *caret* (Kuhn, 2008) and *ggcorrplot* (Kassambara, 2019). Selected variables were centred and normalised and missing values for Lake Mapourika were imputed *via* bagging with the package *caret* before a distance matrix was constructed based on Euclidean distances.

Multivariate differences in the community structure were assessed by a one-way permutational analysis of variance (PERMANOVA; Anderson, 2017) with the factor Ease of human access (4 levels: No access, Walking, Private Access, Public Access). The multivariate analysis was based on Bray-Curtis distance matrices constructed with the *vegan* library (Oksanen et al., 2007) in R and undertaken with 999 permutations. Pairwise comparisons were undertaken using the package *pairwiseAdonis* (Arbizu, 2020) with Benjamini-Hochberg adjusted p values. Differences in the relative abundances of selected taxa depending on Access was undertaken using the Kruskal Wallis test and pairwise comparisons were undertaken with the Dunn Test using Benjamini-Hochberg adjusted p values. Environmental variables (sediment and water column TN, TP and TOC) were tested for significant differences with human access pressure using a Kruskal-Wallis test and Wilcoxon pairwise tests (Benjamini-Hochberg adjusted *p* values).

## Results

### Diversity and composition

Rarefaction analysis showed that across all lakes, ASV richness reached a plateau for planktonic bacteria (Pbact), surface sediment eukaryotes (SSeuks) and surface sediment bacteria (SSbact), suggesting that the sampling effort was sufficient to capture the vast majority of gamma diversity (Fig. S1; Table S8). The number of observed ASVs accounted for greater than 97.5% as predicted by Chao1 for these communities (Table S3). For the planktonic eukaryotes (LPeuks and SPeuks) no plateau was reached indicating diversity levels were underestimated although this was predominantly due to higher diversity in a couple of lakes which did not reach saturation.

**Figure 2:**
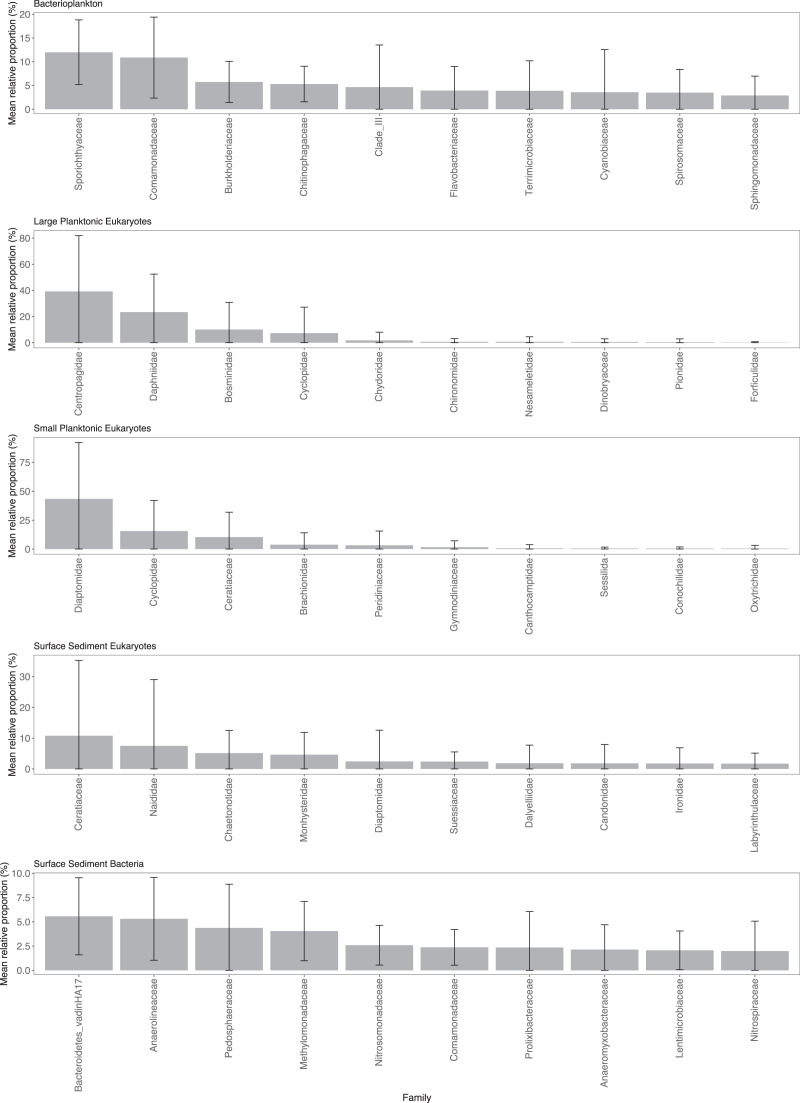
Mean relative abundance of combined Amplicon Sequence Variants (ASVs) at the family level. Only the top 10 families are shown for each community type. Note scale on y-axis varies among community plots.

Sporichthyaceae (mean = 12.0%, sd = 6.8%) and Comamonadaceae (mean = 10.9%, sd = 8.5%) were the dominant components of the Pbact samples (Fig. 2). Copepods were the dominant eukaryotic taxa in the water column with Centropagidae (mean = 39.2%, sd = 42.7%) accounting on average for the highest relative proportion of the LPeuks and Diaptomidae (mean = 43.3%, sd = 48.8%) for SPeuks. In the LPeuks other crustacean taxa, Daphniidae (mean = 23.4%, sd = 29.1%) and Bosminidae (mean = 10.1%, sd = 20.8%) were also important components of the community. In SPeuks, Cyclopidae (mean = 15.6%, sd = 26.4%) and the dinoflagellate Ceratiaceae (mean = 10.3%, sd = 21.6%) accounted for high relative proportions in a range of lakes. The abundances of dominant taxa in the SPeuks and LPeuks were lake dependent with generally a single dominant taxon (Fig. S2). In the SSeuks community, *Ceratium hirundinella* (mean 10.8%, sd = 24.5%) had the greatest average abundance although this was due to high numbers occurring in a small number of lakes (Fig. 2 and Fig. S2). The bacterial family Bacteroidetes vadinHA17 had the highest average abundance across the lakes (mean = 5.6%, sd = 4.0%) in the SSbact followed by Anaerolineaceae (mean = 5.3%, sd = 4.3%) and Pedophaeraceae (mean = 4.4%, sd = 4.5%).

### Assembly processes

**Figure 3:**
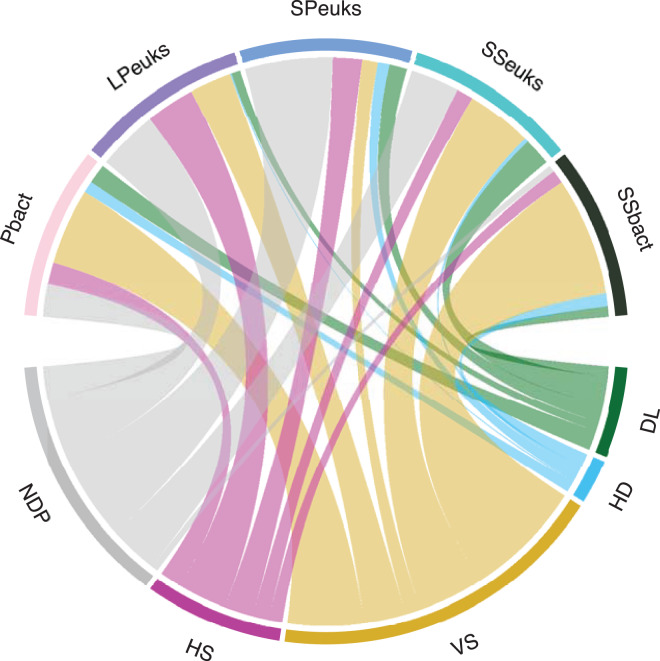
Assembly processes for the different biological components. The width of the bar is proportional to the percent of pairwise comparisons attributed to the assembly process. DL, Dispersal limitation; HD, Homogenizing dispersal; VS, Variable selection; HS, Homogeneous selection. NDP, No Dominant Process; Pbact, bacterioplankton; LPeuks, large planktonic eukaryotes; SPeuks, small planktonic eukaryotes; SSeuks, surface sediment eukaryotes; SSbact, surface sediment bacteria.

Overall, deterministic processes contributed more to the assembly of lake biotic communities compared to stochastic processes (Table 1 and Fig. 3). Deterministic processes were highest in the SSbact accounting for 81% of the comparisons. They were lowest in the SPeuks community making up only about 27% of comparisons with most comparisons (54%) not being dominated by a single process. For the sediment communities (both bacteria and eukaryotes) as well as the Pbact, variable selection was more predominant compared with homogeneous selection while for planktonic eukaryotic communities (LPeuks and SPeuks) the opposite was observed with homogeneous selection contributing more. For the stochastic assembly processes in general, dispersal limitation was more predominant except for SSbact where homogenizing dispersal accounted for a slightly higher proportion of the lake pairwise comparisons. For all biotic components there was a general tendency that lakes that were dominated by homogenizing dispersal were closer together than those where dispersal limitation was predominant. However, this difference was only significant for the Pbact (Kruskal Wallis: chi squared 7.94; *p* = 0.005) and SSeuks (Kruskal Wallis: chi squared 13.92; *p* < 0.001).

**Table 1:** The proportion of lake pairwise comparisons attributed to each assembly process for the different biological components assessed in the study.

	Deterministic		Stochastic		No dominant process
	VS (%)	HS (%)	DL (%)	HD (%)	(%)
Bacterioplankton	46.9	13.5	12.1	7.1	20.3
Large planktonic eukaryotes	25.8	30.3	5.5	0.9	37.4
Small planktonic eukaryotes	9.7	18	11	7.2	54.2
Sediment eukaryotes	40.1	10.2	17.1	2.5	30.1
Sediment bacteria	73.3	8.1	6.4	8	4.2

**Note:**

VS, variable selection; HS, homogeneous selection; DL, dispersal limitation; HD, homogenizing dispersal.

**Figure 4:**
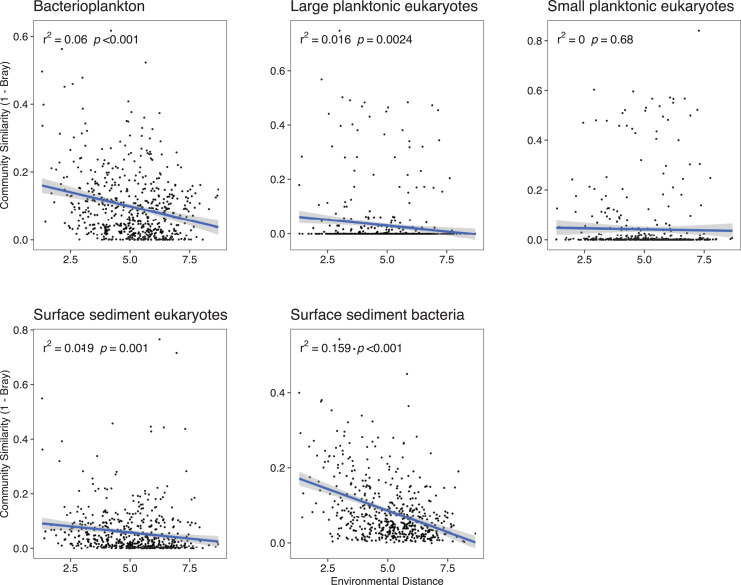
Community similarity (1–Bray-Curtis) against environmental distance for the various community components.

Because deterministic processes were the major component of the assembly processes further investigations of environmental distances *vs* community similarity were undertaken. The bacterial communities showed a higher turnover (slope of the regression line) with increasing dissimilarity in the environment especially in the surface sediments (Fig. 4). With the exception of the SPeuks (*p* = 0.68), significant negative trends were present between community similarity and environmental distance although r^2^ values were low for all communities indicating a weak trend.

### Anthropogenic access pressures

The environmental variables TN, TP and TOC in the water column and sediment were investigated to assess if they varied with human access pressure. There was no significant relationship between human access and sediment TN and TOC (Kruskall-Wallis chi sq = 6.5 and 5.1; *p* values = 0.091 and 0.164 respectively). However, there was a significant difference for sediment TP (*p* = 0.030), although pairwise comparisons were not significant. In the water column all variables were significantly different with human access pressure (TN: chi sq = 9.9; p = 0.019. TP: chi sq = 18.3; *p* < 0.001. TOC chi sq = 13.8; *p* = 0.003.). In general, private lakes had the highest levels of nutrients with pairwise comparisons showing significant differences between private and no access lakes for all water column variables.

**Figure 5:**
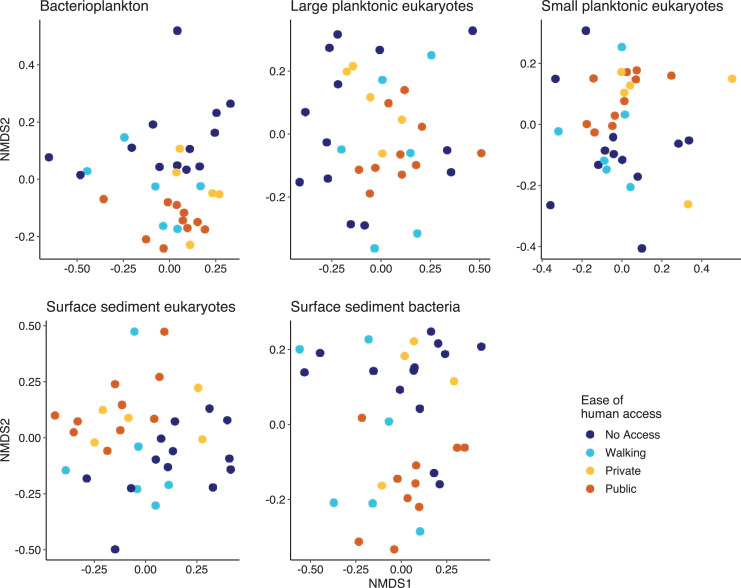
Non metric multidimensional scaling (NMDS) plots for the five biological components. Points are coloured by the ease of human access to the lake.

Multivariate analysis indicated that except for the SPeuks (*p* = 0.078), the community structure of the biological components was significantly different depending on ease of human access (Fig. 5). Pairwise comparisons indicated that differences were mainly related to the comparison between lakes with no access and those with public access (Pbact *p* = 0.006; LPeuks *p* = 0.006; SSeuks *p* = 0.006; SSbact *p* = 0.006). For the LPeuks (*p* = 0.006) there was a significant difference between the communities with private access and those with no access and for the Pbact (*p* = 0.021) and LPeuks (*p* = 0.023) there was a difference between lakes with private access and public access.

Across all the biotic communities there were two taxa that showed strong patterns related to ease of human access. In the Pbact, clade III of the class SAR11 had a significantly different relative abundance (Kruskal-Wallis chi-squared = 15.66, *p* = 0.001; Table 2) in lakes depending on ease of human access. It was not detected in the lakes with no access. It was present in 14 (out of 21) of the lakes with access and had a significantly higher relative abundance in the lakes with public access.

**Table 2:** The relative abundance and standard deviation of clade III of SAR11 and *Daphnia pulex* depending on the ease of human access to the lakes.

	Clade III of SAR11		*Daphnia pulex*	
Ease of human access	Mean relative abundance	Standard deviation	Mean relative abundance	Standard deviation
No access	0	0	0.17	0.62
Walking	4.8	8.31	27.7	22.7
Private	6.43	13.6	0.003	0.003
Public	9.72	10.3	29.2	44

There were also significant differences (Kruskal-Wallis X^2^ = 10.25, *p* = 0.016; Table 2) in the invasive *Daphnia pulex* according to ease of human access with a higher average abundance in the lakes with public access (mean = 29.2%; sd = 44.0%). Pairwise comparisons showed that there was a significant difference between lakes with public access and those with no access (*p* = 0.01). *Daphnia pulex* was only detected in one of the 13 lakes which had no public access. The lowest abundances were observed in the private access lakes where mean abundance was <0.01%.

## Discussion

Our analysis of 34 lakes on the South Island of New Zealand demonstrated that the assembly of biotic communities in both lake water columns and bed sediments was primarily driven by deterministic processes, although the contribution of the various processes differed between biotic components. We also showed that the ease of human access to the lakes had a substantial effect on the biotic communities in the lakes.

### Diversity and community composition

The planktonic bacterial (Pbact) community had high proportions of Sporichthyaceae, within the class Actinobacteria. This group is frequently found to be abundant within lakes across the world (Glöckner et al., 2000; Warnecke, Amann & Pernthaler, 2004; Jiao et al., 2018; Kraemer et al., 2020) and has previously been shown to be an important component in New Zealand lakes (which included the lakes assessed here; Pearman et al., 2022). The small size of the Actinobacteria, may protect them from protistan grazing (Allgaier & Grossart, 2006) which, combined with their ability to degrade a variety of complex organic materials, makes them competitive in lake environments (Ghai et al., 2014). The family Comamonadaceae, class Gammaproteobacteria, was another taxa which contributed substantially to the Pbact and has also previously been shown to be abundant in freshwater environments (Willems, 2014; Pearman et al., 2022).

Zooplankton taxa typical of New Zealand (Parkes & Duggan, 2012; Duggan, Özkundakci & David, 2021), were found to dominate the large planktonic eukaryotes (LPeuks) in the study lakes with the families Centropagidae, Daphniidae (comprised of the genus *Daphnia* and *Ceriodaphnia*) and/or Bosminidae (genus *Bosmina*) especially prevalent. The dominance of different zooplankton species in lakes is likely to have impacts on the food webs and carbon cycling due to distinct size ranges of favoured prey with copepods (Centropagidae) preferring larger sized prey compared to cladocerans (Daphniidae; Burns & Schallenberg, 1996; Sommer et al., 2001). For example, lakes with Centropagidae in high relative abundances did not have high relative abundances of Ceratiaceae (phytoplankton) in the SPeuks. In lakes not dominated by copepods (*e.g*., those with high relative abundances of Daphniidae/Bosinidae), *Ceratium hirundinella* had higher abudnaces. This might be due to lower top-down pressures controlling its abundance thus allowing populations to increase, potentially forming blooms, as has been suggested in Lake Hayes (New Zealand, Schallenberg & Schallenberg, 2017).

In the small planktonic eukaryotes (SPeuks), the copepod family Diaptomidae had the highest relative abundance in most lakes. While Diaptomidae, which are non-indigenous, have been reported in New Zealand lakes (Duggan, Green & Burger, 2006), they are not believed to be widespread in lakes of the South Island. Given the correlation in occurrence between the Diaptomidae in the SPeuks and Centropagidae in the LPeuks we investigated whether the Diaptomidae had been misclassified. Further investigation indicated that there was no 18S rRNA reference sequence belonging to the genus *Boeckella*, which was the dominant Centropagidae taxa in these samples. Thus, this genus could not be correctly assigned using the 18S rRNA gene if present in the SPeuks samples. The potential misclassification of ASVs belonging to the family Diaptomidae highlights the issue of incomplete reference databases, potentially resulting in the wrong conclusions being obtained. Further collaborations between taxonomists and molecular biologists are required to improve reference databases so that they reflect the communities being studied.

The family Ceratiaceae had the highest mean relative abundance in the SSeuk community as well as the third highest in the SPeuks community with the ASVs taxonomically classified as belonging to *Ceratium hirundinella*. This high abundance was mainly driven by the dominance of this species in a restricted number of lakes. This mixotrophic dinoflagellate is known to form problematic blooms in lakes which have been associated with fish kills in New Zealand lakes (Bayer, Schallenberg & Martin, 2008; Schallenberg & Schallenberg, 2017). The high abundance of this species, especially in Lakes Alexandrina, McGregor and Poerua, may indicate that blooms are forming and potentially are impacting food-web interactions (Schallenberg & Schallenberg, 2017) and affecting ecosystem services.

In the SSbact community the abundances of the top ten taxa were more similar with Anaerolineaceae and the environmental group Bacteroidetes vadinHA17 having the highest average abundance. Both are considered to occupy anaerobic habitats and to be involved in the degradation of complex organic matter (Wang et al., 2019).

### Community assembly

The dominance of deterministic processes, especially variable selection, in both the SSbact and SSeuks indicate that differences in the environmental variables present in the surface sediments of the lakes are driving the assembly of these components. This agrees with various studies undertaken on Chinese lakes (Zeng et al., 2019; Jiao et al., 2020, 2021). Sediment DNA includes DNA from organisms living in the sediment, and those that have settled out from the water column or been transported in from the surrounding catchment. The inclusion of these latter two groups will impact the assessment of the processes dominating sediment assemblages. To assess the component actively living in the sediment studies incorporating metabarcoding of the RNA should be undertaken.

In the water column, deterministic processes were still dominant over stochastic processes (although in SPeuks no dominant processes were most important). For the Pbact, variable selection was the most important deterministic process. Other studies have suggested that homogeneous selection is the dominant assembly process in the water column (Zeng et al., 2019; Jiao et al., 2020; Kraemer et al., 2020) although Jiao et al. (2021) showed that dominance of variable or homogeneous selection varies with season. Only a limited number of studies have investigated the structuring of eukaryotic lake communities. In the present study, we found that in general no dominant process was important in the structuring of SPeuks and LPeuks communities. However, homogeneous selection dominated in some instances. Logares et al. (2018), in a study of eukaryotes in Antarctic lakes also showed that a substantial proportion of the lakes had no dominant process (drift acting alone) structuring them. The prevalence of no dominant processes may indicate that other factors such as initial inoculations, may be impacting the structuring of the SPeuks and LPeuks communities.

Previous studies (Jiao et al., 2020, 2021; Xia et al., 2022) have shown that bacterial sediment communities and water column communities were structured differently with homogeneous selection being more prominent in the water column in comparison to the sediment. This pattern was observed for both bacteria and eukaryotes although homogeneous selection was not the dominant process for bacteria in the water column. This pattern suggests that the sediment environment is more heterogeneous than the water column, providing more habitats for organisms and allowing selection to drive more community divergence than would be expected by chance. This is further exemplified with the current results by the turnover (slope of community similarity against environmental distance) in communities being higher for sediment communities compared to those in the water column.

In general, homogenizing dispersal contributed the least to the assembly of the biological communities (except for the SSbact, where it was slightly higher than dispersal limitation). In the current dataset this would be expected as the majority of the lakes studied were not connected and thus dispersal would likely be *via* aerial means (Wilkinson et al., 2012) or by birds (Figuerola, Green & Michot, 2005; Tesson et al., 2018), which may not be sufficiently great enough to allow homogenizing dispersal to be substantially influential in the assembly of the lake communities. It has previously been suggested that dispersal limitation is higher for larger organisms such as zooplankton than phytoplankton and bacteria (Soininen et al., 2011). However, in the current study this was not observed with dispersal limitation accounting for the lowest proportion for LPeuks. However, it has been shown that dispersal limitation is scale dependent (Havel & Shurin, 2004; Soininen et al., 2011), which could indicate that the scale of the current study was not sufficient for dispersal limitation to be more important than local environmental factors in the assembly of the biological communities.

### Anthropogenic access pressures

The lakes in the current study have different levels of human access ranging from being nearly inaccessible (except by helicopter or long off-track trekking) to having easy drivable public access. In the present study, we showed that there was a significant difference in the lake communities (except for the SPeuks) based on human access, with pairwise comparisons indicating the differences were mainly between lakes with no access and those that were easily accessible to the public. One possible reason for this could be differences in the environmental variables based on human access. No clear trend was noted in the sediment geochemistry with access pressure with only TP having a significant difference. Although pairwise comparisons were not significant, in general lakes with private access had higher levels of TP with this likely to be linked to the low productivity grassland in the catchment. In the water column significant differences were noted for TN, TP and TOC with pairwise comparisons indicating that private and public access lakes had higher values, this is most likely linked differences in the catchment. Human alterations of the catchment have previously been shown to impact lake communities (Kraemer et al., 2020). While not measured in this study increased human access would likely result in higher concentrations of contaminants, such as polyaromatic hydrocarbons originating from the combustion of fossil fuels by cars and boats. A small scale metagenomic study of lakes in the South Island of New Zealand showed decreased levels of xenobiotic pathways in a lake surrounded by a conservation zone compared to two lakes that had higher impacts from humans (Biessy et al., 2022).

Noticeable differences were observed at the family level in the distribution of taxa dependent on ease of human access. Most noticeably, the Pbact, clade III of SAR 11 was present in the majority (70%) of the accessible lakes but completely absent from those lakes which were classed as inaccessible. Clade III is a freshwater sister group of the marine group that dominate marine waters and have been found to have a global distribution (Salcher, Pernthaler & Posch, 2011). While widely distributed, they are generally less abundant in the freshwater environments than their marine counterparts (Newton et al., 2011) and thus, have received less scientific interest. The lack of this group in any of the lakes that have no human access is interesting and may indicate that for this group dispersal is naturally limited amongst lakes, with human activities aiding dispersal. However, we acknowledge that other reasons such as the presence of preferable environmental conditions could also explain some of the distribution patterns. While little is known about the ecology of this clade seasonal patterns have been noted, with higher abundance when water temperatures are greater than ~16 °C (Salcher, Pernthaler & Posch, 2011). Most of the lakes with no clade III representation had surface waters with temperatures less than ~16 °C at the time of sampling suggesting that environmental conditions in these lakes may limit the proliferation of this clade. Further work would be required to investigate the environmental niche of this clade and to untangle the drivers that are limiting its distribution in lake systems.

The proliferation of non-indigenous species within lake systems due to human activities has been shown across the world and can have substantial impacts on the ecology of the lakes (Craig, 1992; Mills et al., 1994; Rowe, 2007; Kilroy et al., 2021). *Daphnia pulex* is not native to New Zealand but has spread throughout the South Island of New Zealand since its introduction about 60 years ago (Ye et al., 2021) when it was thought to have been introduced with the stocking of lakes with Chinook salmon (*Oncorhynchus tshawytschca*) (Duggan et al., 2012; Ye et al., 2021). *Daphnia pulex* produces a higher number of ephippia compared to New Zealand native species. Additionally, the ephippia of *D. pulex* float on the water surface for long periods of time until they adhered to surfaces (Burns, 2013). The adherence to boats, fishing gear and other recreational equipment could explain the higher prevalence of this species in the lakes with easy public access. *Daphnia pulex* has the potential to considerably affect pelagic food webs of New Zealand lakes, which are generally species poor (Burns, 2013).

## Conclusions

In this study we used metabarcoding to investigate different biological components in 34 lakes located on the South Island of New Zealand. The aim of our study was to enhance understanding of how lake biotic communities are assembled and distributed. Our results showed that overall deterministic processes dominated the assembly of lake communities. However, the contribution of variable and homogeneous selection differed depending on the biotic component. Variable selection was especially important in the sediment (SSbact and SSeuks) and for the bacterioplankton (Pbact) while homogeneous selection was predominant for the eukaryotic plankton (SPeuks, LPeuks). Our results also showed that human access impacts lake communities. Two exemplars were clade III of SAR11 and *Daphnia pulex*, which were only present in lakes with easy access. With increasing anthropogenic impacts on lakes, increased knowledge on the distribution and assembly processes of the different ecological components in lakes is vital in assisting efforts aimed at mitigating the effects humans have on these ecosystems.

## Supplemental Information

10.7717/peerj.14378/supp-1 Supplemental Information 1 Rarefaction curves showing the number of Amplicon Sequence Variants (ASVs) observed across the total sequencing effort for each biological component of the lakes. Note the different values on x and y axes.

10.7717/peerj.14378/supp-2 Supplemental Information 2 Composition of the different biological components of each lake. Only the top 10 classified families are shown for each community type.

10.7717/peerj.14378/supp-3 Supplemental Information 3 Metadata of the 34 lakes sampled in this study.

10.7717/peerj.14378/supp-4 Supplemental Information 4 Metabarcoding Reveals Lacustrine Picocyanobacteria Respond to Environmental Change Through Adaptive Community Structuring. Primer sequences and PCR conditions used in this study.

10.7717/peerj.14378/supp-5 Supplemental Information 5 SSbact ASV table. Metabarcoding Reveals Lacustrine Picocyanobacteria Respond to Environmental Change Through Adaptive Community Structuring *ASV table for the surface sediment bacteria (SSbact) component*.

10.7717/peerj.14378/supp-6 Supplemental Information 6 SSeuks ASV table. Metabarcoding Reveals Lacustrine Picocyanobacteria Respond to Environmental Change Through Adaptive Community Structuring *ASV table for the surface sediment eukaryote (SSeuks) component*.

10.7717/peerj.14378/supp-7 Supplemental Information 7 SPeuks ASV table. Metabarcoding Reveals Lacustrine Picocyanobacteria Respond to Environmental Change Through Adaptive Community Structuring *ASV table for the small planktonic eukaryote (SPeuks) component*.

10.7717/peerj.14378/supp-8 Supplemental Information 8 LPeuks ASV table. Metabarcoding Reveals Lacustrine Picocyanobacteria Respond to Environmental Change Through Adaptive Community Structuring *ASV table for the large planktonic eukaryote (LPeuks) component*.

10.7717/peerj.14378/supp-9 Supplemental Information 9 Pbact ASV table. Metabarcoding Reveals Lacustrine Picocyanobacteria Respond to Environmental Change Through Adaptive Community Structuring *ASV table for the planktonic bacteria (Pbact) component*.

10.7717/peerj.14378/supp-10 Supplemental Information 10 Alpha diversity in lakes. Metabarcoding Reveals Lacustrine Picocyanobacteria Respond to Environmental Change Through Adaptive Community Structuring *Total number of Amplicon Sequence variants (ASVs) across the lakes and the number of ASVs per lake*.

## Additional Information and Declarations

### Field Study Permissions

The following information was supplied relating to field study approvals (*i.e*., approving body and any reference numbers): All samples were collected under the specifications of Special Permit 651 from the New Zealand government agency Ministry for Primary Industries.

### DNA Deposition

The following information was supplied regarding the deposition of DNA sequences: The sequence reads are available at NCBI SRA: PRJNA750120, PRJNA762383 and PRJNA853061.

### Data Availability

The following information was supplied regarding data availability: The code for the statistical analysis is available at GitHub: https://github.com/jkpearmanbioinf/LakeAssemblyProcesses.
